# Vitamin D Status and Spine Surgery Outcomes

**DOI:** 10.1155/2013/471695

**Published:** 2013-04-11

**Authors:** William J. Rodriguez, Jason Gromelski

**Affiliations:** Nola Physical Therapy, 2 West 45th Street, Suite 208, New York, NY 10036, USA

## Abstract

There is a high prevalence of hypovitaminosis D in patients with back pain regardless of whether or not they require surgical intervention. Furthermore, the risk of hypovitaminosis D is not limited to individuals with traditional clinical risk factors. Vitamin D plays an essential role in bone formation, maintenance, and remodeling, as well as muscle function. Published data indicate that hypovitaminosis D could adversely affect bone formation and muscle function in multiple ways. The literature contains numerous reports of myopathy and/or musculoskeletal pain associated with hypovitaminosis D. In terms of spinal fusion outcomes, a patient may have a significant decrease in pain and the presence of *de novo* bone on an X-ray, yet their functional ability may remain severely limited. Hypovitaminosis D may be a contributing factor to the persistent postoperative pain experienced by these patients. Indeed, hypovitaminosis D is not asymptomatic, and symptoms can manifest themselves independent of the musculoskeletal pathological changes associated with conditions like osteomalacia. It appears that vitamin D status is routinely overlooked, and there is a need to raise awareness about its importance among all healthcare practitioners who treat spine patients.

## 1. Introduction

A large portion of the United States population experiences low back pain, and an increasing number undergo spinal fusion each year. While many patients achieve a satisfactory outcome, there is a subpopulation that fails to achieve acceptable outcomes. These patients may have obvious causes for their outcomes (e.g., pseudarthrosis); however, there are those that are deemed fused yet continue to experience low back pain and other symptoms. Although many factors may be considered when a patient experiences a suboptimal outcome following spinal fusion surgery, serum vitamin D concentration is rarely considered even though most physicians acknowledge its importance in maintaining musculoskeletal health. In this review, we discuss the role of vitamin D in musculoskeletal health especially in relation to low back pain and outcomes of spinal fusion surgery. Our discussion includes the risk factors and prevalence rates of hypovitaminosis D as well as the literature published on vitamin D and spinal surgery. A key concept is the fact that hypovitaminosis D is not asymptomatic, and we delineate the mechanisms by which hypovitaminosis D can adversely affect spinal fusion outcomes.

## 2. Vitamin D

### 2.1. What Is Vitamin D?

The term vitamin D refers to a group of structurally related metabolites [1]. Vitamin D is classified as a fat-soluble vitamin; however, it is not technically a vitamin because the body is capable of producing vitamin D_3_. Structurally, vitamin D is a secosteroid [1]. Individuals obtain vitamin D either from dietary sources, supplementation, or cutaneous synthesis. Vitamin D is hydrophobic, and, therefore, upon entering plasma it is bound by vitamin D binding protein (DBP) [2]. DBP transports vitamin D to the liver where hydroxylating enzymes catalyze the first of two hydroxylation reactions to form 25(OH)D (25-hydroxyvitamin D) [3]. 25(OH)D is eventually transported to the kidney where a second hydroxyl group is added to form 1,25-dihydroxycholecalciferol (1,25-dihydroxyvitamin D or 1,25(OH)_2_D) [4]. The kidney also is capable of producing over 30 other vitamin D metabolites [5].

### 2.2. Vitamin D Nomenclature

Unfortunately, the literature contains a wide array of vitamin D nomenclature for both vitamin D metabolites and vitamin D status, most of which either have no standardized recommendations for use or the common usage does not conform to those recommendations [1, 6]. Matters can be further confounded by the fact that definitions have changed over time and authors can be inconsistent; for example, two orthopedic studies submitted for publication the same year by the same group of authors inexplicably defined vitamin D sufficiency as 30 ng/mL in one publication and as 32 ng/mL in the other [7, 8]. It is beyond the scope of this review to discuss recommended nomenclature versus what is frequently utilized. However, in an attempt to avoid confusion, we have elected to use the terms most commonly found in the literature to describe both vitamin D metabolites and status [9–14].

### 2.3. Determining Vitamin D Status

 The major circulating form of vitamin D, 25(OH)D, is the only metabolite universally recommended to determine the vitamin D status of an individual [6, 15–17]. The most commonly used assays to determine serum 25(OH)D concentrations are high-performance liquid chromatography (HPLC) and radioimmunoassay (RIA). Expert guidelines do not favor either technology; however, studies have demonstrated that HPLC is more sensitive and less variable than RIA when assaying 25(OH)D [6, 17]. Nevertheless, this does not alter the fact that hypovitaminosis D prevalence rates are high regardless of the technology used to assay 25(OH)D concentrations or the concentration used to define vitamin D sufficiency; in fact, Binkley et al. utilized both HPLC and RIA and determined that prevalence rates were substantial by either measurement [17].

### 2.4. Vitamin D Sufficiency

There is consensus that individuals with serum 25(OH)D concentrations less than 20 ng/mL are vitamin D deficient. However, there is disagreement over what 25(OH)D concentration is sufficient; the Institute of Medicine of the National Academies (IOM) recommends 20 ng/mL, while the majority of those specializing in vitamin D research, as well as expert guidelines, recommend concentrations greater than 30 ng/mL [6, 15, 18–22]. Some of the differences of opinion are due to individuals considering only the relationship between one factor and health outcome (e.g., 25(OH)D concentrations and parathyroid hormone levels). However, one must take into account all health outcomes when considering recommendations for sufficient 25(OH)D concentrations. Those specializing in vitamin D research have done this, and they determined that 25(OH)D concentrations should be greater than 30 ng/mL [6, 15, 19–22]. For the purposes of this review, vitamin D sufficiency is defined as a serum 25(OH)D concentration greater than 30 ng/mL [6]; hypovitaminosis D refers to any and all 25(OH)D concentrations below the level of sufficiency (i.e., <30 ng/mL) [9–11]. Finally, whenever possible, the actual 25(OH)D concentrations are provided to facilitate the understanding of what these terms represent.

### 2.5. Prevention and Treatment of Hypovitaminosis D

When considering prevention and treatment of hypovitaminosis D, one must take into account both recommended 25(OH)D concentrations and the recommended dietary allowance (RDA). In terms of the RDA, studies found that up to 46% of individuals meeting or exceeding the 1997–2009 RDA had insufficient 25(OH)D concentrations [16, 23–26]. Experts in the field of vitamin D research, therefore, stated that individuals required a vitamin D intake greater than the 1997 RDA in order to maintain sufficient 25(OH)D concentrations [15, 16, 24, 27–30].

In 2010, the IOM increased the RDA to 600 IU per day for those of 1–70 years of age and 800 IU for those over 70 years of age [18]. Once again those that specialize in vitamin D research, expert guidelines, and published data suggest that this increased RDA still is likely inadequate to ensure that individuals maintain sufficient 25(OH)D concentrations [6, 15, 20, 31–33]. This is especially true for those at increased risk for hypovitaminosis D. However, as discussed below, the risk of hypovitaminosis D extends beyond traditionally defined categories to include the majority of the population. Individuals with hypovitaminosis D require a daily vitamin D intake greater than the RDA, and in fact, the RDA is for healthy individuals only [26, 34, 35]. The recommended treatment for hypovitaminosis D is an eight-week course of either 50,000 IU vitamin D once per week or 6000 IU daily, followed by maintenance therapy of 1500–2000 IU per day [6].

Furthermore, published studies suggest that even healthy adults require a daily vitamin D intake greater than the RDA of 600 IU in order to maintain sufficient 25(OH)D concentrations. For example, several studies suggest that an intake between 700 and 1000 IU per day is needed to maintain sufficient 25(OH)D concentrations [20, 31, 32]. Bischoff-Ferrari et al. suggested an intake of at least 1000 IU daily for all racial-ethnic groups; whereas Heaney estimated that individuals needed close to 2000 IU daily [15, 33]. Finally, the Endocrine Society guidelines state that individuals of 19–70 years of age require at least 600 IU of vitamin D daily and those over 70 years of age require at least 800 IU; however, they also state that all adults over 19 years of age may require at least 1500–2000 IU of vitamin D daily in order to maintain a serum 25(OH)D concentration consistently greater than 30 ng/mL [6].

### 2.6. Vitamin D Safety

 Like any hormone, there is a healthy range for vitamin D levels; concentrations outside this range can lead to health problems. In terms of safety and 25(OH)D concentrations, the IOM recommends that concentrations of 50–60 ng/mL should be avoided [18]. In terms of safety and daily vitamin D intake, both expert guidelines and the IOM set the tolerable upper limits (ULs) of vitamin D intake at 4000 IU per day for adults; this amount should not be exceeded without medical supervision [6, 18]. To summarize, most experts consider 600 IU of vitamin D daily to be the minimum intake, and that to maintain 25(OH)D concentrations of at least 30 ng/mL, an appropriate daily intake is 1500–2000 IU of vitamin D. It should be noted that even though the expert recommendations for both daily vitamin D intake and 25(OH)D concentrations exceed those of the IOM, both fall well within the IOM recommended safety standards.

## 3. Prevalence of Hypovitaminosis D

There is a high-prevalence rate of hypovitaminosis D among the general population. The National Health and Nutrition Examination Survey (NHANES) revealed an overall prevalence rate of approximately 30% using a serum 25(OH)D concentration of 20 ng/mL as a threshold; however, the prevalence rate would have been over 70% if a concentration of 32 ng/mL had been used as the threshold [36]. Studies of healthy children and adolescents reported hypovitaminosis D prevalence rates of 73% (<32 ng/mL) and 45% (≤20 ng/mL), respectively [13, 37], while Binkley et al. found a prevalence rate of 51% (<30 ng/mL) among young adults [38]. Thomas et al. studied general medical inpatients and reported a hypovitaminosis D prevalence rate of 57% [16]. However, the threshold was a serum 25(OH)D concentration of less than or equal to 15 ng/mL; the authors stated that had they used a concentration of 30 ng/mL as the threshold, the prevalence rate would have been 93% [16]. 

Orthopedic patients also have high rates of hypovitaminosis D. Bogunovic et al. studied adults undergoing a variety of orthopedic procedures and found 43% had hypovitaminosis D (<32 ng/mL) [9]. Unnanuntana et al. published both a prospective and a retrospective study of adult total hip arthroplasty (THA) patients and reported hypovitaminosis D prevalence rates of 46.6% (<30 ng/mL) and 39.5% (<32 ng/mL), respectively [7, 8]. Parry et al. conducted a study of pediatric orthopedic patients and found a hypovitaminosis D prevalence rate of 90% (<32 ng/mL); additionally 50% of all patients had 25(OH)D concentrations less than 20 ng/mL [39]. Among female patients with hip fractures, LeBoff et al. found 96% had hypovitaminosis D (≤32 ng/mL) and 85% had 25(OH)D concentrations less than or equal to 20 ng/mL [40]. Brinker et al. studied patients with nonunions and found a hypovitaminosis D prevalence rate of 57% (≤30 ng/mL) [41]. 

There is a paucity of vitamin D-related spine studies. Waikakul found that all nine patients in a group with failed back surgery syndrome had hypovitaminosis D (<30 ng/mL); other case reports of failed spine surgery found similar rates [42–45]. Kim et al. studied female spinal fusion patients and found all 31 had hypovitaminosis D (<30 ng/mL) preoperatively [46]. The largest spinal fusion study to date (313 preoperative patients) revealed a hypovitaminosis D prevalence rate of 57% (<30 ng/mL); furthermore, 27% of patients had 25(OH)D concentrations less than 20 ng/mL [47]. The prevalence of hypovitaminosis D is high among patients with chronic low back pain. One study of 360 chronic back pain patients reported the prevalence of severe vitamin D deficiency (<9 ng/mL) as 83% [48]. Another study of chronic low back pain patients found 82% to have hypovitaminosis D (defined as a serum 25(OH)D concentration between 20 and 40 ng/mL, which is higher than the 30 ng/mL threshold most utilized); although they reported hypovitaminosis D in the control group, none of the controls had a serum 25(OH)D concentration less than 32 ng/mL [10].

There also appears to be a high prevalence of hypovitaminosis D among patients with chronic musculoskeletal pain. A study of patients with persistent musculoskeletal pain found a hypovitaminosis D prevalence rate of 93% (<20 ng/mL) [14], while another reported a prevalence rate of 58% (<20 ng/mL) [49]. Therefore, one can conclude that the prevalence of hypovitaminosis D is incredibly high, whether considering the general population or a specific subset thereof; this holds true even if one uses the most conservative definitions of vitamin D sufficiency (i.e., the lowest 25(OH)D concentrations).

## 4. Risk Factors for Hypovitaminosis D

Those commonly agreed upon to be most at risk for hypovitaminosis D include the elderly, females, obese persons, individuals with dark skin pigmentation, and persons living in northern latitudes and/or that have limited sun exposure. However, multiple studies have demonstrated a high prevalence of hypovitaminosis D in populations that would not be deemed at risk.

### 4.1. Age

 Age is a risk factor for hypovitaminosis D because the elderly have a diminished physiological ability to synthesize vitamin D due to decreased amounts of a vitamin D precursor in their skin [50]. Despite this risk factor, clinical studies have demonstrated that age is not a good predictor of 25(OH)D concentrations. Studies of orthopedic surgery and spinal fusion patients found high-prevalence rates of hypovitaminosis D among younger adult patients [7–9, 47]. One of these studies found that patients of 51–70 years of age were 35% less at risk for hypovitaminosis D compared with those of 18–50 years of age [9]. Thomas et al. looked at a subgroup of medical inpatients with no known risk factors for hypovitaminosis D and found greater than 40% were vitamin D deficient even though the mean age of this subgroup was 44 ± 14 years [16]. The prevalence of hypovitaminosis D among those in a pediatric residency program was 69% even though the mean age was 29.6 ± 2.5 years [51]. Studies focusing on young adults, teens, and children also found prevalence rates of 42–90% [13, 37–39]. Therefore, considering published clinical data, one must conclude that although the elderly are at risk for hypovitaminosis D, the risk extends to individuals of all ages, and that age is not a good predictor of serum 25(OH)D status.

### 4.2. Skin Tone

Skin tone is a risk factor because melanin can absorb UVB radiation; it is estimated that vitamin D synthesis can be reduced by greater than 90% in very dark-skinned individuals [52]. However, clinical studies have demonstrated a high prevalence of hypovitaminosis D among whites [8, 9, 14, 16, 40, 46, 47, 51]. Recent orthopedic and spine studies comprised mainly of white patients had hypovitaminosis D prevalence rates greater than 40% [8, 9, 47]. These are high rates for a population generally not considered to have an increased risk. One must conclude that even though dark-skinned individuals are at risk for hypovitaminosis D, the risk extends to light-skinned individuals as well (albeit for reasons other than skin pigmentation). Therefore, skin tone is not a good predictor of serum 25(OH)D status, and it would be unwise to assume that white patients are not at risk.

### 4.3. Sun Exposure, Seasonality, and Latitude

Those living in northern latitudes during winter and those with limited exposure to sunlight are known to be at risk for hypovitaminosis D [53]. Even among those who have exposure to ideal sunlight, the amount of UVB radiation that reaches the skin varies. Clothing can exclude varying amounts of UVB radiation depending on the style and color, and sunscreen with an SPF as low as 8 can exclude up to 95% of UVB radiation [54, 55]. Binkley et al. found a high prevalence of hypovitaminosis D in individuals with substantial sun exposure even though 40% never utilized sunscreen, and the remainder were outside for at least 20 hours per week without sunscreen [17]. Clinical studies also demonstrated high rates of hypovitaminosis D in lower latitudes, as well as during the summer [17, 39, 41, 56]. Although lack of, or limited, sun exposure certainly puts an individual at risk for hypovitaminosis D, sun exposure regardless of latitude, season, or length of exposure does not ensure an individual to have a sufficient 25(OH)D concentration [17, 39, 41, 56]. Therefore, these sun exposure variables are not good predictors of serum 25(OH)D status. 

### 4.4. Sex

The female sex traditionally has been considered a risk factor for hypovitaminosis D. A few large studies confirm this association [36, 57]. However, some studies found no differences between sexes [14, 39]. Whereas, other studies found that the female sex was associated with a decrease in risk for hypovitaminosis D, and the male sex was identified as a risk factor [8, 9, 58]. In light of these apparently conflicting data, it would be unwise to assume that an individual is not at increased risk based on their sex.

### 4.5. Body Mass Index

Obesity is a risk factor for hypovitaminosis D. Multiple clinical studies confirm that there is an inverse correlation between body mass index (BMI) and 25(OH)D concentrations [8, 9, 13, 59–61]. Bogunovic et al. quantified the risk for orthopedic surgery patients; compared with patients of normal BMI, obese patients were twice as likely to have hypovitaminosis D [9]. Furthermore, the odds of hypovitaminosis D increased by approximately 5% for every unit increase in BMI [9]. However, clinical studies also reveal high rates of hypovitaminosis D among populations that are not obese [51]. Therefore, although the obese are at elevated risk, one should not assume that a nonobese individual is not at risk.

### 4.6. Other Risk Factors

Any physiological condition and/or medication that alters intestinal absorption or vitamin D metabolism can increase the risk of hypovitaminosis D [62]. Stoker et al. found prior cholecystectomy was a predictor of hypovitaminosis D in spinal fusion patients [60]. Counterintuitively, metabolic bone disease may decrease the risk of hypovitaminosis D as was found in a study of orthopedic surgery patients [9]. The authors concluded that this was likely the result of patient awareness of their condition and their being managed with vitamin D supplementation [9].

### 4.7. Predicting Hypovitaminosis D

Published data demonstrate that the risk of hypovitaminosis D is not limited to individuals with traditional clinical risk factors. It is also clear that standard clinical risk factors are poor predictors of hypovitaminosis D. Indeed, Thomas et al. concluded that, at best, they would have been able to identify only 68% of vitamin D deficient patients using risk factors alone [16]. Additionally, in a subgroup of patients with no known risk factors for hypovitaminosis D, 42% were found to have a serum 25(OH)D concentration less than or equal to 15 ng/mL [16]. Since it is difficult to predict hypovitaminosis D by the presence or absence of risk factors alone, the only way to accurately identify this condition is to determine the 25(OH)D concentration of an individual. Holick recommends testing patients once a year to determine their vitamin D status [63]. 

## 5. Musculoskeletal Roles of Vitamin D

Vitamin D has numerous and varied roles in the body, this is underscored by the fact that the vitamin D receptor (VDR) has been identified in several dozen diverse tissues and cells, including those not involved in calcium homeostasis [64]. Two types of VDRs have been identified, and ligand binding to each type activates a distinct signal transduction pathway. The first type of receptor identified was a nuclear VDR (VDR_nuc_). The genomic responses that result from the activation of VDR_nuc_ include transcriptional regulation of specific genes [65, 66]. The second type of receptor identified was a cell surface VDR (VDR_mem_). Activation of the VDR_mem_ results in rapid local responses like the opening of voltage-gated calcium channels and transcaltachia—rapid stimulation of intestinal calcium absorption [67–69]. Studies of VDR knockout mice revealed muscular effects one might not expect like muscle fibers with smaller diameters and postural control abnormalities [70–72]. However, the best-known role of vitamin D is its role in calcium homeostasis; appropriate serum calcium concentrations are essential for many functions, including proper mineralization of the bone, muscle contraction, and transmission of nerve impulses. And studies of VDR knockout mice also revealed the metabolic and skeletal abnormalities one would expect like secondary hyperparathyroidism, hypocalcemia, osteomalacia, and growth retardation [71].

### 5.1. Vitamin D and Bone Tissue

Vitamin D deficiency can result in musculoskeletal pathological changes. The traditional understanding is that vitamin D deficiency eventually results in changes like hypocalcemia and secondary hyperparathyroidism. These changes can lead to the upregulation of osteoclastic activity resulting in resorption of mineral content from bone tissue; prolonged demineralization and the resulting weakening of bone tissue eventually upregulate osteoblastic activity. However, due to the inadequate calcium and phosphate concentrations, the newly formed osteoid is not sufficiently mineralized. Interestingly, recent data indicates that individuals may be vitamin D deficient and have increased bone turnover without having secondary hyperparathyroidism [73]. Previous studies confirmed that hypovitaminosis D increases bone turnover and that individuals may have hypovitaminosis D without secondary hyperparathyroidism [10, 13, 17, 41, 74–76]. Therefore, vitamin D appears to play a direct role in bone formation, maintenance, and remodeling; indeed VDRs have been identified in both osteoblasts and osteoclasts [65, 77–79]. 

 Data reveal that osteoblasts express both nuclear and membrane VDRs [65]. Vitamin D treatment of osteoblasts leads to increased synthesis of bone matrix proteins as well as extracellular signals involved in angiogenesis like vascular endothelial growth factor [65, 80]. Vitamin D also causes a calcium influx into osteoblasts, and this influx is not required for the increased synthesis of bone matrix proteins [65]. Additional data shows that vitamin D-VDR binding in osteoblasts regulates osteoclast formation [81]. There appears to be conflicting data in the literature regarding the role of vitamin D and osteoclasts [78, 82]. However, closer examination of the literature reveals that vitamin D may affect osteoclastic activity in two separate ways. First, the most studied interaction, vitamin D can induce osteoclast formation [81, 82]. This occurs indirectly via vitamin D interaction with osteoblasts, which in turn interact with osteoclast precursor cells. Takeda et al. demonstrated that VDR knockout mice (VDR-*null*) could form osteoclasts [81]. Osteoclasts were formed when osteoblasts and osteoclast precursors, both VDR-*null*, were cultured together and treated with either parathyroid hormone (PTH) or IL-1*α* [81]. However, if osteoblasts and osteoclast precursor cells, both VDR-*null*, were cultured together and treated with vitamin D, osteoclasts were not formed [81]. Finally, when VDR-expressing (wild-type) osteoblasts were cultured with VDR-*null* osteoclast precursors, osteoclast formation occurred with vitamin D treatment [81]. Taken together these data show that osteoclast formation can be induced by multiple, independent signals (i.e., PTH and vitamin D), and that a VDR expressed by osteoblasts mediates vitamin D-induced formation of osteoclasts. There is a second, less studied role of vitamin D in regards to osteoclastic activity—the direct interaction of vitamin D with osteoclasts [77, 78]. Takasu et al. showed that vitamin D could decrease osteoclast formation and that a VDR expressed by osteoclast precursors mediates this activity [78]. Therefore, it appears that vitamin D may regulate osteoclastic activity via two different mechanisms—indirectly (via osteoblasts) and directly. Osteoclastic activity is often thought of in a negative manner; this, however, is not the case unless it is uncoupled from osteoblastic activity and/or inappropriately high. In fact, osteoclastic activity is a necessary part of healthy bone formation and maintenance. 

 One thing is certain, whether via an indirect role (involving PTH), a direct role, or both, vitamin D plays an essential role in bone formation and maintenance. *In vivo* studies on the effect of vitamin D on fracture healing confirm this. Fu et al. investigated the effect of vitamin D on osteoporotic fracture healing in an ovariectomized rat model [83]. Delayed healing occurred in the control group compared to the vitamin D-treated group at 6 and 16 weeks after fracture [83]. This was demonstrated in each of several measures: radiographic (X-rays and micro-CT scans), biomechanical testing, and histological examination. At 6 weeks after fracture, the vitamin D-treated group had a greater amount of bony callus compared to the control group; the vitamin D group also had continuous callus and mature woven bone, whereas the control group had delayed endochondral ossification [83]. Biomechanically, at 6 weeks, the ultimate load at failure and energy absorption were 96% and 95% higher in the vitamin D group, respectively [83]. Histological examination at 16 weeks revealed remodeled mature bone at the fracture site in the vitamin D group, whereas the control group was still undergoing endochondral ossification; both biomechanical measures were higher in the vitamin D group at this time point as well [83]. This *in vivo* study and others demonstrate that vitamin D treatment results in accelerated bone healing and maturation, as well as stronger bone tissue [84, 85]. A randomized placebo-controlled study of proximal humerus fracture healing in women revealed similar results [86]. Unfortunately, the treatment group received both vitamin D and calcium; therefore, one must attribute the results to the combined effect. However, the data is in accordance with vitamin D only treatment in the previously discussed studies. At 6 weeks after fracture, the treatment group had a significantly higher increase in bone mineral density compared to the control group [86]. Finally, hypovitaminosis D-delayed bone formation and healing may account for the findings of Brinker et al. in patients with nonunions [41].

In summary, published data indicate that hypovitaminosis D could adversely affect bone formation in multiple ways: (1) decreasing intestinal calcium absorption thereby limiting calcium availability for both mineralization of newly formed osteoid and cellular functions; (2) altering both the number and activity of osteoblasts and osteoclasts. Although PTH can play a role in each of these effects, vitamin D can affect both independent of PTH. Therefore, in terms of spinal surgery patients, hypovitaminosis D could result in delayed spinal fusion or pseudarthrosis, as well as impaired osseointegration of implanted spinal hardware.

### 5.2. Vitamin D and Muscle Function

Both types of VDRs have been identified in human skeletal muscle cells [87–89]. Vitamin D can directly affect skeletal muscle cells in multiple ways. It affects phosphate transport and phospholipid metabolism; in addition, calcium metabolism and transport is affected at both the genomic and nongenomic levels [90–94]. Vitamin D also promotes muscle cell proliferation, growth, and differentiation [72, 95–97]. Studies of myopathy associated with hypovitaminosis D began appearing in the 1960s and have continued to the present [43, 98–101]. This myopathy has been observed in patients with and without osteomalacia as well as with and without hyperparathyroidism [98, 99, 102–107]. The myopathy is described as muscular atrophy and weakness [98, 100, 101, 103, 105, 106]. Unfortunately, many review articles restrict their description of the muscle weakness to proximal muscle weakness, they often mention altered gait also. However, Mytton et al. reported that proximal muscle weakness and gait change occurred in less than 3% and 2% of patients with this myopathy, respectively [108]. Others reported findings similar to Mytton et al. [10, 109]. Additionally, patients often complain of fatigue [14, 49, 102, 107, 108]. Vitamin D treatment has been shown to improve these symptoms [98, 100, 102, 107, 110–112]. 

In terms of clinical studies of vitamin D and muscle function, some apparently conflicting data have been published. There are reviews solely dedicated to vitamin D and skeletal muscle that address this issue [113]. Nevertheless, both *in vitro* and *in vivo* studies demonstrate that vitamin D plays a direct role in normal muscular function. In fact, human studies have revealed the effect of vitamin D on muscle fibers. Vitamin D deficient adults have been shown to have type II muscle fiber atrophy, and vitamin D treatment increased both the amount and diameter of type II muscle fibers [110, 114]. Two areas are particularly relevant to the outcomes of spine surgery patients in regards to hypovitaminosis D and impaired muscle function: first, trunk stability because spine patients require core strength and stability, especially during the fusion process [115]; second, lower limb weakness because many spine patients have lower limb muscle atrophy. Prolonged weakness in these two areas may lead to extended use of assistance devices (e.g., back braces and canes) [99, 105, 106]. Furthermore, protracted core weakness also may place additional stress on implanted hardware and/or adjacent spinal segments.

### 5.3. Vitamin D and Musculoskeletal Pain

There are numerous reports of musculoskeletal pain associated with hypovitaminosis D [10, 14, 43–45, 48, 49, 102, 103, 107–109, 116–119]. This pain is described as both bone pain and muscular pain [14, 49, 108]. The bone pain is reported as both excluding the joints and including the joints [102, 107, 109]. Often, but not always, the musculoskeletal pain begins in the low back [107–109]. Vitamin D treatment has been shown to improve these symptoms [43–45, 48, 102, 107, 109, 117, 120]. Resolution of symptoms often occurs between three and seven months [102, 107, 109].

The mechanism often proposed to generate hypovitaminosis D-related musculoskeletal pain is that insufficiently mineralized osteoid absorbs fluid and swells; the expanded osteoid then exerts pressure on the periosteum and corresponding nociceptors, thus generating pain [14, 63, 119]. However, Tague et al. recently published neurological data which implicates a different mechanism [121]. First, Tague and Smith identified VDRs on sensory neurons [122]. Next, in a separate publication, they demonstrated that vitamin D deficiency produced muscle hypersensitivity and balance deficits in rats similar to those observed in humans [121]. Vitamin D deficiency resulted in nociceptor hyperinnervation and hypersensitivity that was specific to skeletal muscle tissue; neither occurred in rat hindpaw skin [121]. Interestingly, increased calcium intake exacerbated the adverse effects instead of ameliorating them [121]. Their model was one of early vitamin D deficiency and not prolonged deficiency. Therefore, the pathological features of prolonged vitamin D deficiency were not observed, including altered bone morphology and muscle atrophy [121]. From these data, they concluded that it is unlikely that musculoskeletal pain during early vitamin D deficiency is the result of either skeletal or muscle pathology, rather it is the result of an increased density of pain sensing nerves in muscle tissue [121]. However, as previously discussed, musculoskeletal pathology is known to occur with prolonged hypovitaminosis D. It may be that both of these mechanisms (nociceptor hyperinnervation and musculoskeletal pathological changes like expanded osteoid) work either independently or concomitantly to generate pain in patients with hypovitaminosis D. Finally, in terms of clinical relevancy, both animal and human data show that insufficient 25(OH)D concentrations can produce symptoms in patients prior to and/or independent of the pathological changes that are thought of in connection with vitamin D deficiency [10, 102, 121]. Therefore, hypovitaminosis D is not asymptomatic. 

## 6. Vitamin D and Spine Patients

### 6.1. Spine Studies

Unfortunately, there is a dearth of vitamin D-related spine data, and only four articles have data from 30 or more patients [10, 46–48]. The limited number of spine studies include both spinal surgery patients and those without surgical intervention [10, 42–48]. Two studies examined correlations between pain and 25(OH)D concentrations in patients with back pain. The first study reported that patients with chronic back pain had significantly lower 25(OH)D concentrations compared to a control group [10]. The second, a study of preoperative spinal fusion patients, found that those with vitamin D deficiency had greater pain and higher disability scores [47]. The symptoms described for spine patients with hypovitaminosis D are similar to those discussed in the sections on muscle function and musculoskeletal pain [10, 42–45, 47, 48]. Treatment with vitamin D has been shown to improve these symptoms [42–45, 48]. One study reported that 95% of all patients and 100% of patients with severe vitamin D deficiency had resolution of chronic low back pain three months following vitamin D treatment [48]. Similar results have been reported for patients with chronic back pain and failed spinal fusion surgery [42, 44, 45]. 

Of the two spinal fusion studies with greater than 30 patients, one reported preoperative data only and the other pre- and postoperative data [46, 47]. As previously discussed, Stoker et al. reported an association between vitamin D deficiency and pain and disability scores, as well as a high prevalence of hypovitaminosis D in a study of 313 patients [47]. The second article looked at 31 female spinal fusion patients and found all to have hypovitaminosis D preoperatively and 16% to be vitamin D sufficient 1-year postoperatively [46]. Vitamin D treatment was not part of this study, as the premise was that patients' 25(OH)D concentrations would increase postoperatively due to the increased mobility. However, the premise holds true only for those patients whose 25(OH)D concentration is limited by their level of mobility; increased mobility would most likely involve increased UVB exposure. However, as previously discussed, Binkley et al. demonstrated that abundant UVB exposure does not ensure vitamin D sufficiency [17]. Therefore, even if all patients' UVB exposure significantly increased postoperatively, only a portion would achieve sufficient 25(OH)D concentrations. And, the data revealed this, only 16% of patients had sufficient 25(OH)D concentrations after 1 year; the remaining 84% still had hypovitaminosis D and, therefore, were at risk for musculoskeletal symptoms like pain, myopathy, and delayed fusion or pseudarthrosis [46]. A correlation between postoperative 25(OH)D concentration and Oswestry disability index (ODI) scores was observed [46]. Unfortunately, 1-year postoperative fusion status data was not reported. 

Even though there is limited spine data, several conclusions can be drawn. First, there is a high prevalence of hypovitaminosis D in patients with back pain regardless of whether or not they require surgical intervention. Second, spine patients with hypovitaminosis D display symptoms similar to other populations with hypovitaminosis D, and the correlation between pain and 25(OH)D concentration also holds true for spine patients. Third, in the absence of an intervention with vitamin D supplementation the majority of spine patients will not achieve sufficient 25(OH)D concentrations after a surgical intervention. Finally, patients with hypovitaminosis D may experience delayed fusion or pseudarthrosis and/or a return of persistent pain that requires revision surgery and/or vitamin D treatment [42–45].

### 6.2. Surgical Outcomes

Although not spinal fusion studies, two recent articles on THA and vitamin D status should be addressed [7, 8]. These studies looked at functional milestones in-hospital and at 6 weeks postoperatively [7, 8]. Neither study found an association between 25(OH)D concentration and functional outcomes. However, the functional outcomes were bare minimum measures and, therefore, would not likely reveal differences between vitamin D sufficient and insufficient patients. The main item these studies reveal is that there are different perspectives of functional outcomes and surgical success; although a patient may qualify as having a technically successful outcome by certain measures, their actual functional ability may be severely limited.

 Often successful spinal fusion surgery is defined by the appearance of *de novo* bone tissue on plain radiographs. However, using X-rays (whether static or dynamic) to evaluate the status of a fusion site has severe limitations. First, surgical implants make evaluation difficult. Second, bone grafts (both natural and synthetic) can be mistaken for *de novo* bone formation [123, 124]. Spinal fusion studies demonstrate that sites deemed fused via X-rays actually contained large amounts of fibrous tissue when assessed via CT scan and/or histology [123–125]. Therefore, patients may be deemed fused without being fully fused. Additionally, successful spinal fusion surgery may be defined as a significant decrease in pain and disability scores (e.g., ODI and SF-36). Unfortunately, there is a subpopulation of spinal fusion patients that are considered technically successful in this regard yet are wholly unsuccessful in terms of their residual pain and disability. Although considered a success, these patients require ongoing treatment for their pain and may continue to have extensive limitations on their mobility and ability to sit, perform weight-bearing activities, and/or return to work. We think (based on the data discussed in this review) that hypovitaminosis D may be a major contributing factor to the symptoms experienced by these patients.

### 6.3. Vitamin D Status Is Overlooked

Published data reveals that physicians routinely neglect evaluating the vitamin D status of a patient. A study of patients with musculoskeletal pain for at least one year found that although greater than 90% had been medically evaluated for their chronic pain, none had been tested for hypovitaminosis D [14]. Upon testing, the prevalence rate for this group was determined to be 93%; furthermore, five of the patients had undetectable 25(OH)D concentrations [14]. Apparently many spine surgeons regularly overlook vitamin D status and underestimate its importance as well. One survey of orthopedic surgeons and neurosurgeons who treat spine conditions found that metabolic bone laboratories (vitamin D, PTH, and calcium) were utilized by 12% and 20% of spine surgeons as part of a workup for preoperative fusion and pseudarthrosis patients, respectively [126]. Furthermore, 33% of those who do not routinely check metabolic bone laboratory tests stated that, “they did not think it would affect clinical management” [126]. Interestingly, of those surveyed, 71% had completed a spine fellowship, and 32% were affiliated with an academic institution [126]. Publications by those who have utilized vitamin D to treat patients with chronic pain due to failed spine surgery confirm that surgical intervention, pain management, and/or physical rehabilitation often are considered while vitamin D status is an afterthought at best [42–45]. Therefore, there is a need to raise awareness about the importance of vitamin D status among all healthcare practitioners who treat spine patients.

## 7. Conclusion 

Given the high prevalence of hypovitaminosis D among the general population as well as spine patients and that it is difficult to predict its presence based on risk factors alone, all spine surgery patients should be screened as part of their preoperative workup. Postoperative spine patients that continue to experience musculoskeletal pain should be screened as well. Patients with hypovitaminosis D should be treated according to the Endocrine Society guidelines discussed above, especially since vitamin D treatment is safe and the cost burden low. Furthermore, hypovitaminosis D is not asymptomatic, and symptoms can manifest themselves independent of (or prior to) the musculoskeletal pathological changes associated with conditions like osteomalacia. Published data indicate that hypovitaminosis D could adversely affect bone formation and muscle function in multiple ways. For spinal fusion patients the consequences may include delayed fusion or pseudarthrosis, impaired osseointegration of implanted spinal hardware, prolonged core, and lower limb muscle weakness, and additional stress on implanted hardware and/or adjacent spinal segments. Any of these, as well as hypovitaminosis D-induced neurological changes, may contribute to a spinal fusion patient's persistent postoperative pain; indeed, musculoskeletal pain is a hallmark of hypovitaminosis D. Therefore, it is imperative for spinal fusion patients to maintain sufficient serum 25(OH)D concentrations (i.e., >30 ng/mL). Finally, although hypovitaminosis D is associated with a multitude of adverse health outcomes, vitamin D is not a panacea but rather one critical factor in maintaining musculoskeletal health.
